# Urologic Involvement in the Management of Ureteral Hernias: A Case Series

**DOI:** 10.1155/criu/9002319

**Published:** 2026-06-22

**Authors:** Sajya M. Singh, Maya Weerasooriya, Joseph Baiocco, Kendrick Campbell, Tova Weiss, Gregory P. Murphy

**Affiliations:** ^1^ Division of Urologic Surgery, Washington University in St. Louis School of Medicine, St. Louis, Missouri, USA, wustl.edu

**Keywords:** distal ureteral reimplantation, inguinal hernia, inguinoscrotal hernia, ureteral hernia, ureteral stent

## Abstract

In this case series, we describe our approach to the urologic management of ureteral hernias. This is a single‐institution, multi‐surgeon cohort of patients who presented with an abdominal hernia involving a ureter between 2018 and 2025. Among eight patients, seven male patients (87%) had an inguinal hernia involving the ureter. Six patients underwent urologic intervention at the time of inguinal hernia repair, most commonly preoperative ureteral stent placement (40%). Three patients (50%) experienced inguinal ureteral hernia recurrence, of whom two underwent ureteral reimplantation. Based on our experience, the majority of inguinal ureteral hernias can be managed with a preoperative ureteral stent at the time of hernia repair, although more complex ureteral reconstruction may be required in the setting of recurrence.

## 1. Introduction

Ureteral hernias occur when part of the ureter protrudes through musculoskeletal foramina with or without other abdominal contents. Overall, this is a rare phenomenon with less than 200 cases documented in the literature [1]. Ureteral hernias most commonly occur in the inguinal or inguinoscrotal region, although there are reports of obturator, femoral, diaphragmatic, and sciatic ureteral hernias [1, 2]. Inguinal ureteral hernias are usually identified intraoperatively at the time of hernia repair or after intraoperative injury [3, 4]. A preoperative diagnosis allows for a multispecialty surgical approach with consideration for ureteral protection given the high risk of injury in the setting of unrecognized ureteral involvement [3, 5]. We aim to add to the limited literature by describing one of the largest case series of ureteral hernias with an emphasis on preoperative evaluation and corresponding intraoperative management.

## 2. Case Presentations

This is a single institution, multi‐surgeon retrospective study of eight patients who were found preoperatively to have abdominal hernias involving a ureter between 2018 and 2025 (Table 1). The patients in this case series were identified due to preoperative consultation to urology by a general surgeon for assistance in the management of a hernia known to involve the ureter. Although we did not seek to exclude cases identified intraoperatively, we are not aware of an intraoperative or postoperative diagnosis involving a urology consultation for an inguinal ureteral hernia at our institution during our study timeframe.

**Table 1:** Patient demographics and perioperative approach to management of ureteral hernias with focus on recurrence and operative outcomes.

ID	Sex	Age	BMI	Prior hernia repair?	Stent?	ICG?	Recurrence?	Outcome
1	M	65	24.1	Y	N	N	—	Hospice
2	F	80	26.0	N	Attempted	N	—	Hospice
3	M	66	44.7	N	Y	N	N	Reduced
4	M	67	37.3	Y	Y	Y	N	Reduced
5	M	73	32.0	Y	Y	N	Y	Not reduced
					Y	Y	N	Reimplanted
6	M	59	32.6	N	Y	N	Y	Reduced
					Attempted	N	Y	Reduced
					Y	Y	N	Reimplanted
7	M	66	49.8	N	N	N	N	Nephrectomy
8	M	68	37.3	N	N	N	Y	Unknown
					Y	Y	N	Reduced

Among eight patients, the seven male patients (87%) had an inguinal or inguinoscrotal hernia, and the one female patient had a sciatic hernia. The first patient in the series was managed nonoperatively. We next describe the perioperative course of the remaining seven patients who were managed with either a procedural or surgical intervention.

### 2.1. Patient 1

Patient 1 was a 65‐year‐old (BMI 24.1 kg/m^2^) Black male with a history of seven hernia surgeries over the preceding 20 years, who presented with worsening scrotal swelling. CT scan demonstrated a large right inguinal hernia involving the distal right ureter, which was associated with right moderate hydronephrosis (Figure 1A). Labs showed a mild acute kidney injury (AKI). The patient transitioned to hospice shortly after diagnosis without intervention.

**Figure 1:**
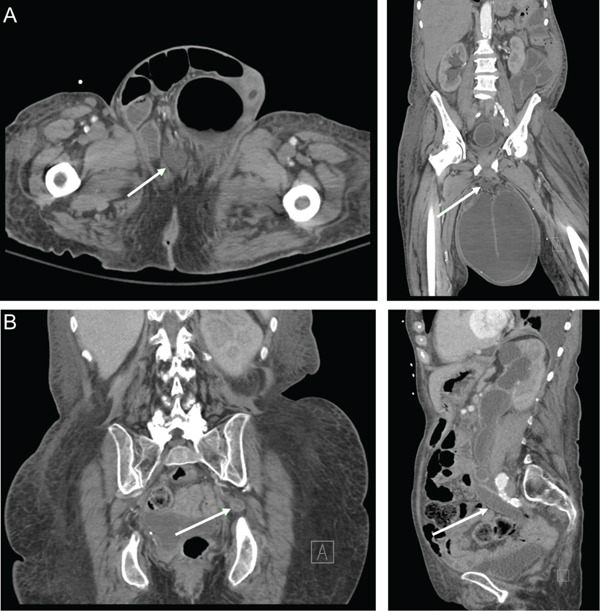
Diagnostic CT scans of Patients 1 and 2. (A) Patient 1′s large right inguinal hernia involving the distal right ureter with associated moderate hydronephrosis. (B) Patient 2′s left distal ureteral hernia through a defect in the sciatic notch with associated moderate hydronephrosis.

### 2.2. Patient 2

Patient 2 was an 80‐year‐old overweight (BMI 26 kg/m^2^) White female, who presented with a urinary tract infection (UTI). CT scan revealed herniation of the distal left ureter through a defect in the sciatic notch with moderate hydronephrosis (Figure 1B). Labs demonstrated AKI. Retrograde ureteral stent placement was unsuccessful. A nephrostomy tube was therefore placed and later converted to an internal ureteral stent. The patient declined further surgical intervention and elected for hospice shortly thereafter.

### 2.3. Patient 3

Patient 3 was a 66‐year‐old morbidly obese (BMI 44.7 kg/m^2^) White male, who presented with painful right inguinal swelling. CT scan revealed severe right hydroureteronephrosis and multiple loops of bowel within the hernia sac (Figure 2A). There was no associated AKI. He underwent preoperative placement of a right 5‐French (Fr) open‐ended ureteral stent followed by an open inguinal hernia repair with successful reduction of the ureter. The stent was removed at the end of the case. Postoperative imaging at 2 months did not show evidence of hernia recurrence.

**Figure 2:**
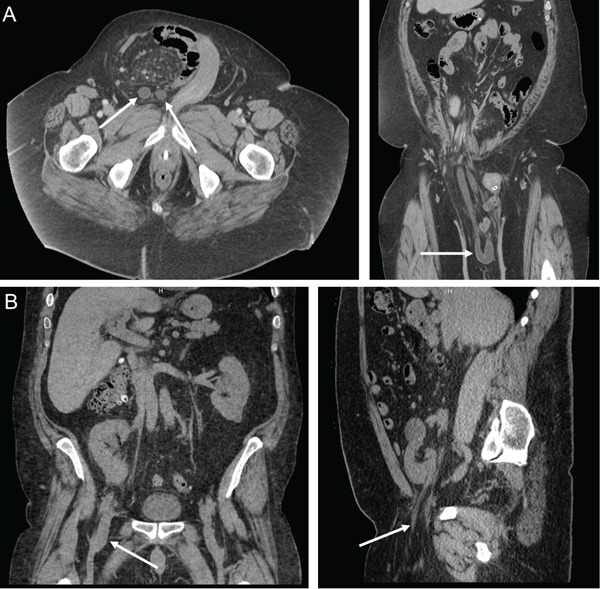
Preoperative CT scans of Patients 3 and 4. (A) Patient 3′s large right inguinal hernia involving the right ureter with associated severe hydronephrosis. (B) Patient 4′s large right inguinal hernia involving the right ureter with associated mild hydronephrosis.

### 2.4. Patient 4

Patient 4 was a 67‐year‐old obese (BMI 37.3 kg/m^2^) White male with a history of previous bilateral open inguinal hernia repair, who presented with right groin pain. CT scan revealed a right pelvic kidney with mild hydronephrosis secondary to the right ureter extending into a large inguinal hernia (Figure 2B). There was no associated AKI. He underwent robotic inguinal hernia repair with preoperative placement of a right single‐J ureteral stent and injection of indocyanine green (ICG) to assist in identification of the ureter. The ureter was successfully reduced, and the stent was removed at the end of the case. No immediate postoperative complications were noted, and the patient was subsequently lost to follow‐up without postoperative imaging available for review.

### 2.5. Patient 5

Patient 5 was a 73‐year‐old obese (BMI 32 kg/m^2^) White male with a pertinent history of prior bilateral inguinal hernia repairs, who presented with flank and abdominal pain in the setting of a mechanical fall. CT scan showed mild right hydroureteronephrosis with the ureter entering the inguinal canal and looping back outwards to the bladder (Figure 3A). There was no associated AKI. He first underwent right hernia repair, which involved preoperative placement of a 5Fr single‐J stent by urology; however, the ureter was unable to be safely reduced, and the stent was removed at the end of the case. He underwent a second attempt at hernia repair 6 months later. After a 7Fr lighted ureteral stent was placed preoperatively, he underwent robotic right inguinal hernia repair. ICG was used intraoperatively for ureteral identification. Again, the ureter was unable to be safely reduced, and the decision was made to perform a right extravesical ureteral reimplant. His postoperative course was uncomplicated, and his hernia had not recurred on imaging 7 months postoperatively.

**Figure 3:**
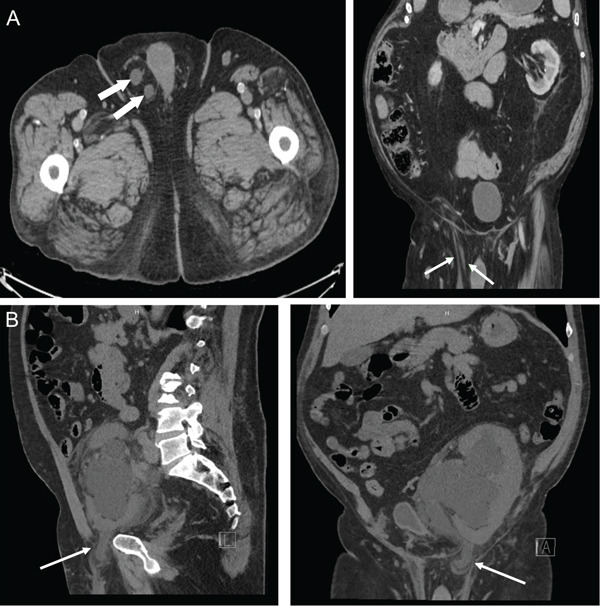
Preoperative CT scans of Patients 5 and 6. (A) Patient 5′s right inguinal ureteral hernia associated with mild hydronephrosis. (B) Patient 6′s transplant ureteral hernia through the left inguinal canal with associated severe hydronephrosis.

### 2.6. Patient 6

Patient 6 was a 59‐year‐old obese (BMI 32.6 kg/m^2^) White male with a history significant for end stage renal disease secondary to hypertensive nephrosclerosis, for which he underwent kidney transplant 30 years prior. He presented with decreased urine output and significant AKI. CT demonstrated severe hydronephrosis secondary to transplant ureter herniation into the left inguinal canal (Figure 3B). He underwent nephrostomy tube placement, which was later converted to an internal ureteral stent. A transplant surgeon then performed an open hernia repair and ureterolysis with successful reduction of the incarcerated hernia and ureter. He had a stent free period between the first and second surgery with stable hydronephrosis and kidney function.

Six months after, he underwent an open inguinal hernia repair for recurrent hernia. Urology was unsuccessful at preoperative stent placement, although the ureter was ultimately successfully reduced during the repair. Five years later, he again had recurrence of his inguinal hernia. His final operation was a robotic hernia repair with ureterolysis and injection of ICG. The decision was made intraoperatively to proceed with robotic transplant ureteral reimplantation. Postoperatively, he developed an AKI and UTI with imaging demonstrating mild transplant hydronephrosis after stent removal. He therefore underwent replacement of a ureteral stent. No hernia recurrence was observed on postoperative imaging performed 5 months later, although he had persistent moderate hydronephrosis after stent removal. The patient required dialysis starting 4 months after his final operation due to persistent kidney dysfunction, likely secondary to expected gradual nephrosclerosis and not due to obstruction per nephrology.

### 2.7. Patient 7

Patient 7 was a 66‐year‐old morbidly obese (BMI 49.8 kg/m^2^) White male with no history of prior hernia repair who presented with bilateral groin pain and swelling. CT scan revealed bilateral inguinal hernias with herniation of the left ureter into the left inguinal canal (Figure 4A). He also had multiple renal stones within a left atrophic kidney (17% function) and a left distal ureteral stone. This was associated with an AKI. Urology performed a robotic left simple nephrectomy concurrently with an open left inguinal hernia repair by general surgery. No immediate postoperative complications were noted.

**Figure 4:**
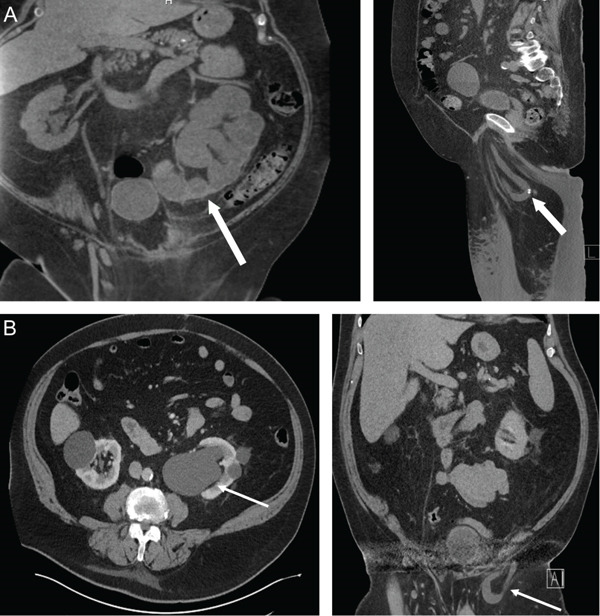
Preoperative CT scans of Patients 7 and 8. (A) Patient 7′s left inguinal hernia involving the left ureter with distal ureteral stones as well as cortical thinning and severe hydronephrosis of the left kidney. (B) Patient 8′s recurrent left inguinal ureteral hernia with associated left hydronephrosis.

### 2.8. Patient 8

Patient 8 was a 68‐year‐old obese (BMI 37.3 kg/m^2^) White male with a known left inguinal hernia, who presented with new urinary urgency and frequency. CT scan demonstrated severe left hydroureteronephrosis extending into a left inguinal hernia. There was no associated AKI. The patient underwent an initial robotic hernia repair at an outside institution with general surgery. No operative records were available for review. A repeat CT scan 12 months postoperatively showed recurrence of left hydroureteronephrosis from ureteral herniation (Figure 4B). He then underwent a second robotic hernia repair with a urologist present. Urology performed cystoscopy with instillation of ICG via an open‐ended ureteral catheter. Later in the case, ureteroscopy was performed given concern for ureteral stricture, which enabled visualization of the ureter intra‐abdominally and assisted with successful reduction. No immediate postoperative complications were noted, and postoperative imaging at 1 month showed no evidence of recurrence.

## 3. Discussion

Ureteral involvement in abdominal hernias is a rare but increasingly described phenomenon in the literature that poses unique challenges for definitive surgical management. Preoperative diagnosis allows for the planned presence of a urologist during hernia repair given that unrecognized ureteral involvement in a hernia poses a high risk for iatrogenic ureteral injury [3, 5]. In our cohort of preoperatively identified inguinal ureteral hernias, there were no intraoperative ureteral injuries. It is important for urologists to understand the risk factors that lead to ureteral herniation, common presentations, and surgical options.

By 2009, there were approximately 140 cases of inguinal ureteral hernias reported, most commonly in kidney transplant patients. These can be paraperitoneal or extraperitoneal, although the majority of cases in the literature (80%) are paraperitoneal with attachment of the ureter to the posterior peritoneum [5, 6]. Here, we highlight prior studies that have used their data in addition to the available literature to provide an approach for patients with an inguinal ureteral hernia. An algorithm proposed by Yahya et al. [6] recommends urinalysis and kidney function tests for select clinical inguinal hernias followed by stepwise imaging for diagnosis. Isernia et al. [7] synthesized 22 articles to propose the Madrid 2021 classification of inguinal ureteral hernias with the goal to grade prognosis and operative risk. There remains no prospective data to inform best practice or to evaluate if published algorithms enable increased rates of preoperative diagnosis, which is needed although challenging given the low incidence of inguinal ureteral hernias.

In our case series, four patients (50%) presented with laboratory results and/or symptoms indicating urinary tract pathology that were distinct from the traditional presentation of inguinal hernias with groin and/or scrotal swelling and pain. Yahya et al. [6] recommended preoperative CT urogram for inguinal hernia patients with unexplained acute renal failure or UTI. Prior publications have also stated that lower urinary tract symptoms in a hernia patient should prompt a CT urogram to assess possible ureteral or bladder involvement [7, 8]. Given the rarity of this diagnosis, we do not recommend routine cross‐sectional imaging for all inguinal hernia patients, which is in line with the prior literature [9]. This case series does offer additional evidence that in patients with inguinal hernias who also have AKI, UTI, urinary symptoms, or flank pain, preoperative abdominal and pelvic cross‐sectional imaging should be obtained to evaluate for ipsilateral hydronephrosis. Ipsilateral hydronephrosis indicative of ureteral involvement was present in all patients in our cohort.

Previously identified risk factors for ureteral involvement in inguinal hernias include male sex, obesity, older age (5th and 6th decade of life), and history of renal transplant [3, 6, 10, 11]. Keeping with the literature, the majority of our cases occurred in obese men in the 6th decade of life (mean age 68 ± 6.3 years, mean BMI 36.2 kg/m^2^). Only one of our eight cases involved a transplant ureter, which is unusual based on prior reports [7, 11]. It is known that when a native ureter is involved, the right side is more commonly herniated, likely due to increased adherence of the left ureter to the retroperitoneum [6]. In our series, following this trend, 57% of cases in a native kidney occurred on the right side.

Scarring and adhesions from prior hernia surgery were proposed as a risk factor for subsequent ureteral involvement in a recurrent inguinal hernia by Golgor et al. [12]. In our series, 38% of patients had a history of inguinal hernia repair prior to ureteral herniation. The proportion of hernia repairs done for recurrent inguinal hernias varies from 2%–3% to 10.5% in reports [13, 14]. Overall, this may indicate that a portion of recurrent inguinal hernia patients are at risk for ureteral involvement, although this finding should be validated in a larger study population.

The most common urologic intervention during the 10 surgical hernia repairs among the six operatively managed patients in our cohort was preoperative ureteral stent placement (*n* = 4, 40%). This is consistent with prior reports that most patients with ureteral hernias will undergo cystoscopy and preoperative ureteral stent placement [15]. Additionally, ICG was used in four cases (40%) to assist with ureteral identification. Use of ICG for ureteral localization has previously been demonstrated to be safe and effective in minimally invasive surgery [16]. To date, this strategy has not been well described in ureteral hernia repair and represents an area of future study [17]. One patient underwent nephrectomy for an atrophic kidney with stone disease, which was a unique scenario in which ureteral reduction was not attempted given known poor kidney function.

Finally, three patients (50%) experienced inguinal hernia recurrence with ureteral involvement after surgical repair. All cases of inguinal hernia recurrence had concomitant ureteral herniation. Ureteral reimplantation was performed in two recurrent inguinal ureteral hernia repairs during which the ureter was unable to be safely reduced. This approach has been rarely previously described [18, 19]. Distal reimplantation was performed with minimal bladder manipulation that did not necessitate use of a Boari flap or psoas hitch. Specific factors that prevent safe ureteral reduction and therefore may lead to the use of ureteral reconstruction in inguinal ureteral hernias require further study.

Based on the experience provided by this case series, we propose an algorithm for the perioperative urologic management of inguinal ureteral hernias (Figure 5). We aim to provide a useful framework for urologists and general surgeons faced with a patient with an inguinal ureteral hernia; however, it must be acknowledged that this algorithm is limited by the lack of validation outside our institution. The algorithm builds on prior schemas aiming to assist in preoperative diagnosis by proposing when to use novel operative strategies, including ICG and ureteral reimplantation.

**Figure 5:**
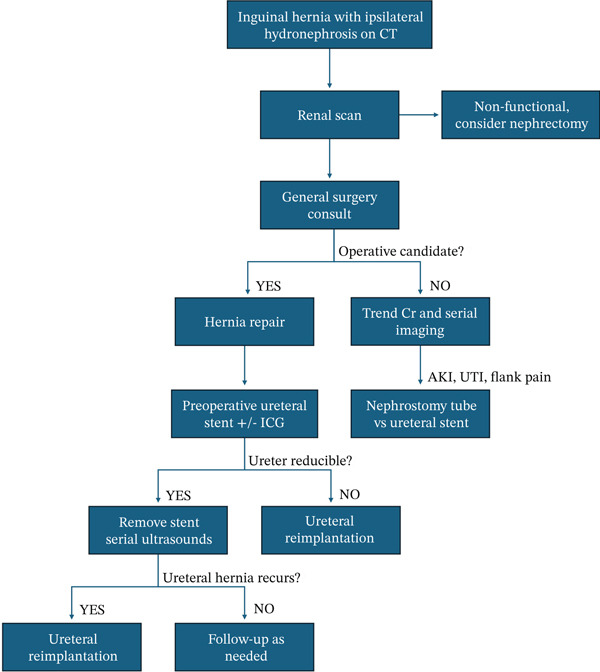
Algorithm for the proposed approach to perioperative urologic management of inguinal ureteral hernias.

In conclusion, new urinary symptoms, AKI, UTI, or flank pain in a patient with an inguinal hernia should raise suspicion for ureteral involvement and prompt preoperative cross‐sectional imaging for further evaluation. Preoperative diagnosis of ureteral involvement in an inguinal hernia allows for planned ureteral protection and the presence of a urologist during surgical repair. The most common urologic intervention in our case series was the placement of a preoperative ureteral stent for assistance in ureteral identification and reduction. Use of ICG and ureteral reimplantation as novel strategies in inguinal ureteral hernia repair represent areas of future study.

## Funding

No funding was received for this manuscript.

## Conflicts of Interest

The authors declare no conflicts of interest.

## Data Availability

The data that support the findings of this study are available from the corresponding author upon reasonable request.
